# A New Strategy for Quality Evaluation and Identification of Representative Chemical Components in* Polygonum multiflorum* Thunb.

**DOI:** 10.1155/2017/6238464

**Published:** 2017-01-24

**Authors:** Yun-xia Li, Xiao-hong Gong, Mei-chen Liu, Cheng Peng, Peng Li, Yi-tao Wang

**Affiliations:** ^1^Institute of Chinese Medical Sciences, State Key Laboratory of Quality Research in Chinese Medicine, University of Macau, Macau; ^2^Pharmacy College, Chengdu University of Traditional Chinese Medicine, State Key Laboratory Breeding Base of Systematic Research, Development and Utilization of Chinese Medicine Resources, Chengdu 611137, China

## Abstract

*Polygonum multiflorum* Thunb. (HSW) is widely used as herb medicine and health food additive. Recently, a series of HSW-induced hepatotoxicities have been reported and many studies have been carried out to investigate it. But contradictory conclusions were drawn that might be caused by the inconsistent quality of market decoction pieces. Therefore, the HSW decoction pieces quality was evaluated with a developed novel method in the paper. 25 batches of raw HSW (RHSW) and 21 batches of processed HSW (PHSW) samples were purchased from different provinces of China. HPLC determination was performed to identify and detect the contents of 16 chemical compounds in herbal material. Fingerprint similarity was analyzed using chromatography information and the results showed that most herbs were in good similarity. Then, a comprehensive evaluation strategy based on principal component analysis with representative quality control indicators was developed to evaluate the quality of HSW samples. And the rationality of the developed method was verified by HCA analysis. The results showed that the herb from Dabashan, Sichuan Province, no matter RHSW or PHSW had the best quality. Different representative components were selected for RHSW or PHSW decoction pieces which might be caused by the chemical reaction during processing. And most PHSW were unqualified according to the requirement of Chinese Pharmacopeia which might take the responsibility for the toxicity of HSW.

## 1. Introduction

The last decade has witnessed an emergence and rapid shift of the paradigm in chemotherapy, involving a gradual transition from monosubstance therapy that had long been advocated with great vehemence to multidrug therapy. As a result, herbal medicines (HMs) are widely used which contain multiple active constituents that synergistically act to elicit effects greater than individual components. The wide application brings great opportunities to HMs but it is also accompanied with big challenges in quality standard for the complex composition.

There are two trends in HMs quality evaluation. For a long time, the content detection of constituents is the main quality control method for HMs that marker compounds are chosen followed by qualitative and quantitative analysis. Therefore, the sample quality is easily to be classified as “qualified” or “unqualified” when the analytical data is directly compared with the presetting criteria. However, the selection of indicators and their content levels of the existing quality standard are often subjective or sometimes a little arbitrarily without powerful evidences [1, 2]. With the development of modern analytical instrument, systematic evaluation emerges to include component information as much as possible [3, 4]. Although the identification tells us what chemical compounds are contained in HMs, it is still hard to distinguish herb quality with such information.

**Figure 1:**
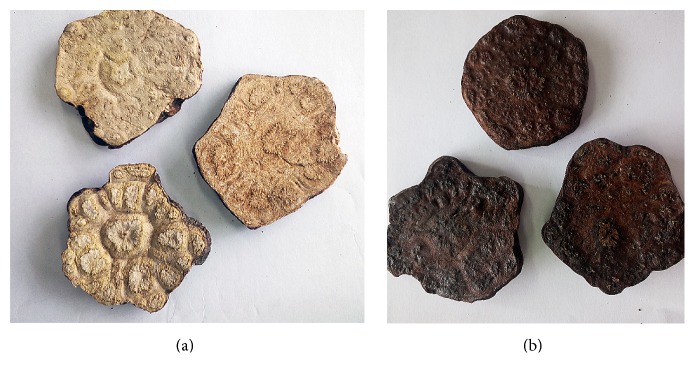
Picture of RHSW (a) decoction pieces and PHSW (b) decoction pieces.

*Polygonum multiflorum* Thunb. (He Shou Wu in Chinese pinyin, herein after referred to as HSW), the root of* Polygonum multiflorum*, is a famous traditional Chinese medicine and is also widely used in East Asia and North America with the name of Fo-ti. Modern pharmacological studies have shown that HSW has the effect of reducing blood lipid, protecting liver, enhancing immunity, improving memory, protecting nerve cells, antioxidation, antiaging, etc. [5]. With the significant biological and pharmacological effects, HSW has been used not only as a drug but also as a food supplement for many centuries in China. Two forms of HSW decoction pieces are available in market (Figure 1). One is raw HSW (RHSW) used for detoxification, eliminating carbuncle, preventing malaria, and relaxing bowel. Another is processed HSW (PHSW) used for nourishing liver and kidney, supplementing essence and blood, blackening hair, strengthening bones and muscles, eliminating dampness, and reducing lipid.

In 2002, RHSW and PHSW were authorized as health food additives by the government of People's Republic of China. Then, both of them were taken as tonic to prevent hair loss and premature graying in varies forms [6, 7]. However, a series of HSW-induced hepatotoxicities were reported in China, Korea, Japan, Britain, Italy, Australia, and other countries [8–10]. As a result, cautions on the intake of HSW preparations have been issued in China, Australia, Britain, Japan, and Canada.

However, people purchased the herb material directly from the market in most reported toxicity cases. The absence of quality control of market decoction pieces might be a key factor influencing HSW toxicity. As we know, various factors including geographical origin, harvesting, and processing influence herb quality which further results in different pharmacological effects [11, 12]. Therefore, the comprehensive chemical composition analysis and sample classification are of great importance for the quality evaluation of HMs which has so far been multivariate and multi-index. To deal with the issue, an innovative comprehensive evaluation strategy based on principal component analysis with representative quality control indicators selection was developed to evaluate HSW quality. This approach of integrating the information of multiple indexes and using a comprehensive approach to evaluate the quality of HSW is an effective way to solve the problems of differences in the weights of index components and interactions among the components.

## 2. Materials and Methods

### 2.1. Chemicals and Reagents

Acetonitrile, methanol, formic acid, and water were of HPLC grade and purchased from Fisher Chemicals (Pittsburg, PA, USA). Gallic acid, procyanidins B_1_, catechin, gallate, aloe emodin-8-*β*-o-glucoside, polydatin, stilbene glycosides, rhaponticin, resveratrol, emodin-8-*β*-o-glucoside, physcion-8-*β*-o-glucoside, aloe emodin, rhein, chrysophanol, emodin, and physcion were purchased from National Institute for Food and Drug Control (Beijing, China) with purities above 98%.

### 2.2. Plant Materials

25 batches of RHSW and 21 batches of PHSW samples (the data were listed in the Supporting Information Table S1, in Supplementary Material available online at https://doi.org/10.1155/2017/6238464) were purchased from different provinces of China. All RHSW and PHSW samples were authenticated as the roots of* Polygonum multiflorum* Thunb. by Professor Jin Pei, and their voucher specimens were deposited at the Herbarium Center of Chengdu University of Traditional Chinese Medicine.

### 2.3. Preparation of Standard and Sample Solutions

16 reference compounds were accurately weighed separately using Sartorius BP 211D electronic balance (Lower-Saxony, Germany) and dissolved in 10 mL volumetric flask with methanol to prepare stock solutions (gallic acid 131 *μ*g/mL, procyanidins B1 98 *μ*g/mL, catechin 185 *μ*g/mL, gallate 142 *μ*g/mL, aloe emodin-8-*β*-o-glucoside 78 *μ*g/mL, polydatin 125 *μ*g/mL, stilbene glycosides 5400 *μ*g/mL, rhaponticin 142 *μ*g/mL, resveratrol 94 *μ*g/mL, emodin-8-*β*-o-glucoside 750 *μ*g/mL, physcion-8-*β*-o-glucoside 86 *μ*g/mL, aloe emodin 71 *μ*g/mL, rhein 81 *μ*g/mL, chrysophanol 500 *μ*g/mL, emodin 25 *μ*g/mL, and physcion 88 *μ*g/mL). Then, different volumes of stock solutions were mixed together to prepare serials of standard solutions with methanol (Table S2).

The samples powder (each about 0.25 g, *n* = 3) was soaked in 5 mL 50% aqueous ethanol for 30 min and then extracted by ultrasonication for 60 min at 40°C. After being filtrated through a 0.45 *μ*m membrane filter, 5 *μ*L was injected for analysis.

### 2.4. HPLC Analysis

The analysis was performed using an Agilent 1260 series HPLC (Agilent) equipped with a quaternary pump, vacuum degasser, autosampler, and diode array detector. Chromatographic separation was carried out on an Agilent Zorbax XDB-C_18_ column (5 *μ*m, 4.6 mm × 250 mm). The mobile phase consisted of acetonitrile (A) and water containing 0.1% formic acid (B) with a linear gradient elution at a flow rate of 1.0 mL/min. The gradient program was as follows: 5~10% A (0~5 min); 10~22% A (5~30 min); 22~25% A (30~38 min); 25~32% A (38~48 min); 32~45% A (48~55 min); 45~85% A (55~65 min); 85~95% A (65~70 min); and 95% A (70~72 min). The detection wavelength was 275 nm. The column temperature was maintained at 30°C.

### 2.5. Method Validation

The detection wavelength was selected by DAD according to the number and height of peaks in the chromatograms of RHSW and PHSW extracts. The calibration curves were fitted to linear regression with a correlation coefficient (>0.999) during the tested concentration ranges. The intra-assay and interassay precision were investigated by injecting standard solutions (three concentrations) six times in a day or in the consecutive three days. Reproducibility was studied through six independently prepared samples from a single batch of HSW. The stability test was performed by successively injecting the same sample solution over 24 hours.

### 2.6. Data Analysis

The chromatogram correlation coefficients among samples were calculated using the software “Similarity Evaluation System for Chromatographic Fingerprint of Traditional Chinese Medicine” (version 2004A) which was recommended by State Pharmacopeia Committee of the People's Republic of China. Principal component analysis (PCA) using Pareto Scaling method in preprocessing data and hierarchical cluster analysis (HCA) using Median clustering method in preprocessing data were performed by SPSS 21.0 (SPSS Inc., Chicago, USA).

Then, a novel evaluation strategy based on PCA with representative quality control indicators was developed to evaluate the quality of HSW samples. After PCA analysis was carried out on the detected chemical components, comprehensive index- integrated *F* value (*F*_*i*_) was calculated as follows:

(1) $$\begin{array}{c} A_{tf}=\frac{B_{tf}}{\sqrt{\lambda_{f}}}, \end{array}$$

where *t* referred to chemical component in the herb; *f* referred to principal component; *A*_*tf*_ was the contribution of component *t* to principal component *f*; *B*_*tf*_ was standardized component *t* contribution to principal component *f*; *λ*_*f*_ was eigenvalue of principal component *f*; and

(2) $$\begin{array}{c} F_{if}=\sum \left(\;A_{tf}\times C_{it}\;\right), \end{array}$$

where *C*_*it*_ was the concentration of chemical compound *t* in sample *i*; *F*_*if*_ was the value of principal component *f* in sample *i*.

The integrated *F*_*i*_ value can be described as follows:

(3) $$\begin{array}{c} F_{i}=\sum \left(\;F_{if}\times V_{f}\;\right), \end{array}$$

where *V*_*f*_ was variance of principal component to the total value.

The weight factor (*W*_*t*_) of component to the whole herb quality was calculated as follows:

(4) Wt=∑Atf×Vf,W=∑Wt,% of weight=WtW×100%.

## 3. Results and Discussion

### 3.1. HPLC Method Validation

To gain high sensitivity and good peak capacity, the chromatographic conditions were optimized. Acetonitrile/0.1% formic acid was used as the mobile phase to improve the retention behavior of the constituents on the HPLC column.

**Figure 2:**
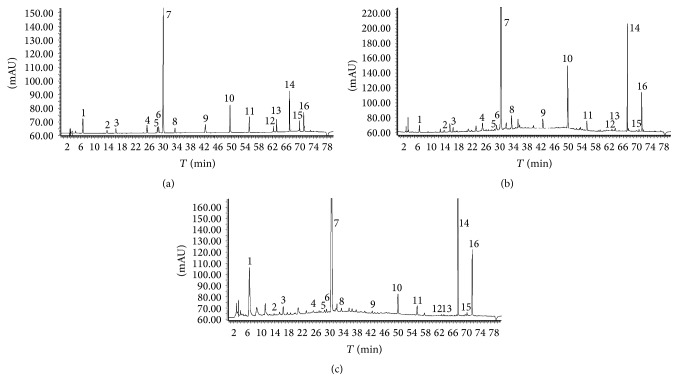
Chromatography of standard solution (a), RHSW (b) decoction pieces and PHSW (c) decoction pieces ((1) gallic acid, *t* = 6.62 min; (2) procyanidins B_1_, *t* = 13.71 min; (3) catechin, *t* = 16.28 min; (4) gallate, *t* = 25.16 min; (5) aloe emodin glycoside, *t* = 28.57 min; (6) polydatin, *t* = 28.94 min; (7) stilbene glucoside, *t* = 30.31 min; (8) rhaponticin, *t* = 33.62 min; (9) resveratrol, *t* = 42.49 min; (10) emodin glycoside, *t* = 49.74 min; (11) physcion glycoside, *t* = 55.31 min; (12) aloe emodin, *t* = 62.35 min; (13) rhein, *t* = 63.48 min; (14) emodin, *t* = 67.13 min; (15) chrysophanol, *t* = 70.17 min; (16) physcion, *t* = 71.33 min).

To detect more components at the same time, full scan was performed to optimize the wave length. The pretest results showed that both peak area and peak number at 275 nm provided more comprehensive profiles of RHSW and PHSW extracts. The relative standard deviations (RSDs) of the peaks were less than 2.0% for precision and 1.96% for reproducibility. The RSDs of the RPAs were less than 1.90% for stability. The recovery for the sixteen standards were 91.56~109.58%. All results indicated that the method was adequate and applicable (Tables S2~S5). Based on the above validated method, 16 constitutes chromatograms of RHSW and PHWS were visually distinguishable from each other and all peaks were simultaneously eluted within 72 minutes (Figure 2). The chemical constitute contents in RHSW and PHWS were listed in Tables 1 and 2.

### 3.2. Preparation of Sample Solutions

To extract as more components as possible, different factors were investigated in pretest including extraction solution (0, 20%, 40%, 50%, 60%, 80%, 100% ethanol), extraction volume (3, 4, 5, 6, 7 mL), soak time (10, 20, 30, 40, 50 min), extraction temperature (20, 40, 60, and 80°C), and extraction time (10, 20, 40, 60, and 80 min). Finally, the optimization extraction solution was as follows: 0.25 g samples powder was soaked in 5 mL 50% aqueous ethanol for 30 min and then extracted by ultrasonication for 60 min at 40°C.

### 3.3. The Quality Evaluation of RHSW

**Figure 3:**
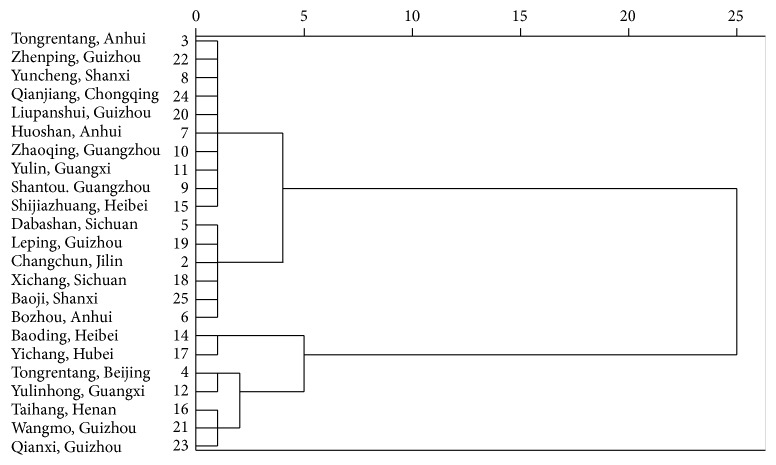
HCA analysis of RHSW decoction pieces with 16 components.

Similarity Evaluation System for Chromatographic Fingerprint of TCM was widely applied in evaluating herb quality by calculating correlation coefficients based on peak area and retention time. The fingerprint similarities of RHSW were shown in Table 3 which ranged from 0.953 to 0.999. Two exceptions were the herbs collected from Kunming city, Yunnan Province (number 1), which had a correlation coefficient of 0.48, and those from Yancheng city, Jiangsu Province (numbers 13, 0.67). The two samples were excluded in HCA analysis which then classified 23 batches RHSW into five groups as in Figure 3.

However, the compounds concentration varied no matter in RHSW or PHSW (Tables 1 and 2) even if herb met similarity evaluation requirement of lager than 0.9. It was impossible to evaluate the herb quality with so much information. HCA can classify the herb into different groups, but the result still cannot give us a conclusion which group had the best quality and so many chemical constitutes observed in the herb which can be chosen as the main components of the herb.

On the basis of chromatography, a two-step systematic strategy was developed to comprehensively evaluate the herb quality and select the representative compounds for the herb. PCA was a multivariate data analysis method to summarize massive numbers of variables in a dataset into a few correlated variables. In the study, PCA was firstly performed on the pretreated HPLC spectra of all RHSW samples. The PCA results showed that original sixteen variables (chemical compounds) were reduced to 6 principal components reflecting 82.33% of the influence of each compound on herb quality (Table 4). The contributions of each compound to principal components were listed in component matrix (Table 5).

The integrated *F* value calculated according to “Materials and Methods” was shown in Table 6 which was in agreement with HCA classification. Group one (S14, S17) had the integrated *F* value of 53.61~85.62; group two (S4, S12) had the *F* value of 117.52~141.01; group three (S167, S21, S23) had the integrated *F* value of 152.61~184.01; group four (S3, S7, S8, S9, S10, S11, S15, S20, S22, S24) had the integrated *F* value of 193.14~228.93; group five (S2, S5, S6, S18, S19, S25) had the integrated *F* value of 244.71~310.43. HCA result verified the reasonable of PCA analysis and indicated the integrated *F* value can be used as index to evaluate the quality of RHSW.

In Chinese Pharmacopoeia (2015 edition), stilbene glycosides content in RHSW decoction was officially set as no less than 1.0% and the sum of the content of emodin-8-*β*-o-glucoside and physcion-8-*β*-o-glucoside was no less than 0.05%.  Table 1 showed that stilbene glycosides content in RHSW samples 1, 13, and 17 was 0.02%, 0.03%, and 0.79%, which was below the standard. The contents of emodin-8-*β*-o-glucoside and physcion-8-*β*-o-glucoside in RHSW samples 1, 4, 7, 8, 9, 12, 13, 16, and 17 was 0.01, 0.03, 0.04, 0.04, 0.03, 0.02, 0.01, 0.02, and 0.02%. Sample S5 (integrated *F* value 310.43) contained the highest stilbene glycosides (2.20%) and emodin-8-*β*-o-glucoside and physcion-8-*β*-o-glucoside content (1.64%) which also verified the conclusion that the integrated *F* can represent the whole herb quality.

**Figure 4:**
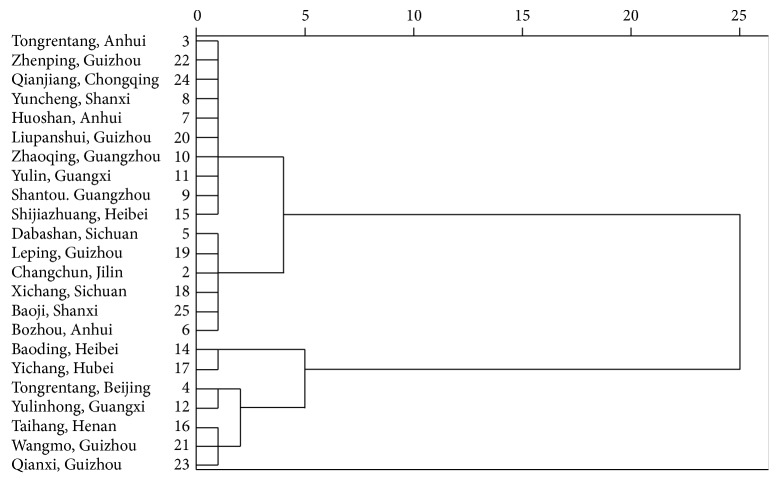
HCA analysis of RHSW decoction pieces with selected 8 representative components.

Although so many chemical compounds were detected in RHSW, which of them contributed most to the herb quality was still unknown. The peak area of components was used to present the contribution in some reports. However, the responsivity of each component was different at the same absorption length. Therefore, the same concentration might induce different peak area which caused error in calculation. In PCA, the data was firstly standardized to eliminate data error. The (% of weight) value of each chemical compound was shown in Table 7. Component with high value (% of weight) was chosen until the cumulative value achieved 80%. In RHSW analysis, eight components were chosen including catechin, stilbene glycosides, rhaponticin, resveratrol, emodin-8-*β*-o-glucoside, rhein, emodin, and physcion. Then, a second HCA of the herb was processed on the chosen 8 components (Figure 4). The analytical data was in accordance with the first HCA made with 16 components which verified the conclusion that the chosen chemicals can present the quality of the whole herb.

In previous herb quality control, the chemical with higher contents tended to be chosen as marker components. However, herb usually contained chemical components with similar structure. During the chemical synthesis, the contents of similar categories were correlated with each other. Therefore, it was unnecessary to analyze all chemicals in quality control. In the paper, the original 16 chemicals were simplified to 8 chemicals. The inclusion of physcion may be a reason for the exclusion of physcion-8-*β*-o-glucoside in the representative components.

### 3.4. The Quality Evaluation of PHSW

The same strategy was also applied to analyze the quality of 21 batches of PHWS samples from market. Fingerprints similarity of PHWS showed that 5 batches are below the required 0.9 (Table S6). Five principle components were selected which presented 81.25% information of PHSW (Table S7). The contributions of each compound in PHSW to principal components were listed in component matrix (Table S8). HCA analysis classified 21 batches of PHSW into four groups as in Figure S1. As shown in Table S9, PHSW group one (S1, S3, S11, S15, S18) had the integrated *F* value of 6.50~36.52. Group two (S4, S5, S10, S16, S19, S20) had the integrated *F* value of 59.79~102.84; group three (S6, S7, S8, S9, S13, S14, S17, S21) had the integrated *F* value of 108.10~141.32; group four (S2, S12) had the integrated *F* value of 175.15~191.12. Sample 12 form Dabashan, Sichuan Province, had the highest *F* value of 191.12 which suggested it had the best quality. The interesting thing was that the best herb was from the same productive area (Dabashan, Sichuan province) no matter RHSW or PHSW which indicated the geography may be more suitable for HSW production.

Six components were chosen as the quality indicators for PHSW: aloe emodin-8-*β*-o-glucoside, polydatin, stilbene glycosides, emodin-8-*β*-o-glucoside, physcion-8-*β*-o-glucoside, and aloe emodin. The chosen components were not the same as those of RHSW. Then, a second HCA of the herb was processed on the chosen components (Figure S2). The analysis result was in accordance with the first HCA which verified the conclusion that the chosen components can present the quality of PHSW.

As for PHSW decoction, stilbene glycoside content was officially set as not less than 0.7% and the sum of the content of emodin and physcion was no less than 0.1%. However, the concentration determination results indicated that stilbene glycoside in samples 1, 3, 11, 15, and 18 failed to meet the Chinese Pharmacopeia requirement with *s* contents of 0.472%, 0.13%, 0.28%, 0.08%, and 0.64%. Stilbene glycosides in samples 2 and 12 were higher than 2.0%. And only the content of emodin and physcion in samples 2, 8, and 12 satisfied the standard of no less than 0.1% with 0.17%, 0.22%, and 0.11%. All the other samples had the content of 0.01%~0.06% which was much lower than 0.1%.

The situation may be caused by two factors. One reason might be the poor RHSW herb quality. But the result of quality evaluation suggested most RHSW quality was good enough to satisfy the requirement of Chinese Pharmacopeia. Another factor was processing technology in preparing PHSW. Both stilbene glycosides and anthraquinone compounds were unstable during processing. Long processing time resulted in the chemical change. In other reports, PHSW was always prepared for a long time, which not only resulted in the decreased content of stilbene glycosides and anthraquinone compounds but also induced the generation of hydroxymaltol, DDMP, and 5-HMF through Maillard reaction [13].

In summary, the study provided insights into the chemical profiles of RHSW and PHSW from different geographical origins. Systematic evaluation of RHSW and PHSW was successfully carried out after multistep data filtering and multivariate statistical analysis. Also, the proposed strategy integrating analysis might be a promising approach for the selection of representative component in the quality evaluation of HMs. Although the developed strategy was carried out on the identified chemical components in the study, it can also be applied in unidentified herb analysis to screen the useful information.

## Supplementary Material

The supporting materials were showed in supplementary tables and figures. The various productive areas of Raw Polygonum multiflorum Thunb. and Processed Polygonum multiflorum Thunb. were included in Table S1. The method validation results of the established HPLC method in detecting various components in Polygonum multiflorum Thunb. were showed in Table S2~5. Also, the calculation results in relation to Processed Polygonum multiflorum Thunb. were showed in Table S6~10. In addition, HCA analysis of Processed Polygonum multiflorum Thunb. decoction pieces with 16 components were showed in Figure S1 and HCA analysis of Processed Polygonum multiflorum Thunb. decoction pieces with selected 6 representative components were showed in Figure S2.

## Figures and Tables

**Table 1:** The content of 16 components in 25 batches of RHSW (*μ*g/g).

Number	Gallic acid	Procyanidins B_1_	Catechin	Gallate	Aloe-emodin glycoside	Polydatin	Stilbene glucoside	Rhaponticin	Resveratrol	Emodin glycoside	Physcion glycoside	Aloe emodin	Rhein	Emodin	Chrysophanol	Physcion
(1)	118.35 ± 2.86	ND	2090.78 ± 14.65	378.99 ± 141.11	110.23 ± 9.82	312.55 ± 48.83	159.20 ± 7.53	31.40 ± 9.05	ND	79.77 ± 10.63	24.08 ± 3.67	ND	ND	70.66 ± 0.09	9.95 ± 0.81	81.76 ± 0.97
(2)	180.11 ± 4.21	209.16 ± 2.20	1029.42 ± 20.44	310.26 ± 0.60	61.36 ± 3.09	180.15 ± 1.28	36355.70 ± 180.43	359.58 ± 0.52	97.40 ± 1.25	1791.22 ± 5.89	235.51 ± 0.61	24.94 ± 0.91	11.96 ± 0.66	485.86 ± 18.12	4.31 ± 2.07	489.68 ± 112.51
(3)	149.60 ± 2.69	26.57 ± 8.06	1142.83 ± 21.02	72.26 ± 9.45	91.85 ± 8.48	225.60 ± 24.03	23441.69 ± 1384.04	241.13 ± 1.24	42.10 ± 7.68	799.75 ± 19.97	291.91 ± 3.09	33.59 ± 1.05	10.34 ± 1.14	551.18 ± 15.21	3.61 ± 0.63	429.86 ± 107.11
(4)	145.30 ± 5.60	37.12 ± 3.47	541.21 ± 81.07	159.75 ± 30.82	151.12 ± 3.69	298.22 ± 11.92	16918.54 ± 4.12	70.63 ± 23.26	62.53 ± 11.98	319.05 ± 5.68	151.19 ± 8.22	20.91 ± 1.29	11.65 ± 0.24	960.99 ± 19.59	4.53 ± 0.13	460.86 ± 85.58
(5)	107.43 ± 6.96	106.45 ± 10.78	851.87 ± 160.88	287.30 ± 17.19	108.62 ± 12.69	277.66 ± 3.65	43982.58 ± 995.13	1180.41 ± 44.82	311.32 ± 12.64	3234.45 ± 19.88	463.20 ± 13.38	59.93 ± 3.07	35.86 ± 3.46	1287.06 ± 10.23	4.58 ± 0.17	656.47 ± 26.84
(6)	144.84 ± 1.78	131.88 ± 11.62	886.66 ± 164.59	428.59 ± 19.39	176.49 ± 28.73	406.71 ± 22.28	39961.24 ± 275.88	1217.68 ± 8.72	283.36 ± 18.43	1340.38 ± 0.05	518.66 ± 10.07	70.05 ± 4.63	71.20 ± 17.28	831.46 ± 29.96	5.25 ± 0.31	447.32 ± 147.98
(7)	113.04 ± 2.17	67.60 ± 6.71	1209.75 ± 0.30	1341.53 ± 2.55	247.48 ± 2.14	768.28 ± 0.50	34682.02 ± 11.72	116.97 ± 39.80	31.64 ± 0.86	355.78 ± 1.28	46.07 ± 13.76	3.27 ± 0.93	1.02 ± 0.28	430.58 ± 3.99	0.84 ± 0.12	200.44 ± 47.98
(8)	521.71 ± 6.67	24.56 ± 0.72	1012.07 ± 10.69	368.22 ± 6.56	196.42 ± 0.35	553.35 ± 20.91	32241.93 ± 163.61	210.73 ± 76.04	149.94 ± 2.10	318.38 ± 32.90	68.69 ± 2.53	34.67 ± 2.24	19.32 ± 2.36	1086.09 ± 35.97	2.70 ± 0.09	452.25 ± 134.65
(9)	257.22 ± 4.30	24.56 ± 4.88	1157.58 ± 18.65	48.48 ± 10.63	40.87 ± 1.48	96.51 ± 0.97	30817.13 ± 106.32	56.08 ± 11.14	21.32 ± 0.76	236.62 ± 0.64	89.48 ± 0.74	65.05 ± 2.93	5.37 ± 0.20	780.91 ± 13.05	3.45 ± 0.10	341.91 ± 61.11
(10)	270.27 ± 2.28	18.48 ± 7.07	972.55 ± 9.02	52.62 ± 12.51	25.52 ± 6.87	83.16 ± 14.01	29921.86 ± 276.39	40.14 ± 22.10	10.75 ± 4.34	1052.58 ± 4.92	184.90 ± 4.05	14.10 ± 0.37	1.25 ± 0.63	119.22 ± 2.36	1.43 ± 0.29	58.33 ± 16.45
(11)	156.80 ± 5.75	102.55 ± 12.99	1077.00 ± 30.35	271.17 ± 6.62	102.49 ± 5.76	213.12 ± 20.05	29916.29 ± 1318.82	250.93 ± 12.44	44.72 ± 9.27	767.30 ± 6.45	221.19 ± 4.28	43.16 ± 2.65	0.79 ± 0.04	591.26 ± 9.50	1.40 ± 0.93	314.87 ± 60.69
(12)	379.20 ± 1.58	105.49 ± 29.03	3868.47 ± 5.94	294.57 ± 1.31	113.58 ± 1.74	329.78 ± 1.32	18439.29 ± 53.49	277.03 ± 15.03	6.98 ± 0.45	145.34 ± 51.13	47.12 ± 1.50	23.12 ± 0.33	2.66 ± 0.06	196.32 ± 0.52	2.67 ± 0.06	117.65 ± 0.68
(13)	9.89 ± 2.13	28.72 ± 4.26	17.65 ± 2.11	123.36 ± 4.88	13.81 ± 0.24	27.87 ± 0.47	270.00 ± 9.53	26.28 ± 2.23	ND	42.81 ± 0.99	20.83 ± 0.78	ND	45.71 ± 0.34	96.58 ± 13.54	49.20 ± 3.80	6.65 ± 1.23
(14)	133.63 ± 3.79	189.75 ± 10.63	2130.11 ± 1.49	379.23 ± 161.46	227.30 ± 4.74	458.82 ± 26.90	10161.47 ± 12.66	318.34 ± 16.97	46.36 ± 2.80	543.26 ± 39.78	40.05 ± 6.21	10.37 ± 5.40	84.12 ± 2.17	160.47 ± 41.76	7.02 ± 0.60	198.21 ± 11.29
(15)	41.54 ± 1.79	70.34 ± 4.42	320.35 ± 38.40	168.54 ± 5.41	56.62 ± 9.48	128.03 ± 12.03	29750.90 ± 611.85	404.95 ± 15.74	13.96 ± 1.26	1883.54 ± 7.26	497.61 ± 3.54	51.1 ± 27.53	8.74 ± 0.34	653.58 ± 12.59	2.78 ± 0.71	435.42 ± 82.68
(16)	142.47 ± 4.04	158.79 ± 5.63	566.41 ± 10.35	71.00 ± 13.97	26.25 ± 4.53	53.44 ± 6.14	24400.56 ± 1452.61	44.85 ± 37.42	18.47 ± 6.77	208.21 ± 2.82	124.82 ± 3.13	101.16 ± 8.87	3.50 ± 0.19	555.02 ± 15.72	4.84 ± 1.07	508.66 ± 21.96
(17)	233.16 ± 2.85	49.48 ± 8.63	0.60 ± 0.22	39.47 ± 4.10	26.22 ± 8.48	72.51 ± 3.80	7895.54 ± 60.82	43.36 ± 1.46	2.36 ± 1.00	203.70 ± 1.90	129.82 ± 2.21	5.53 ± 2.00	21.20 ± 2.86	441.27 ± 6.77	2.86 ± 0.94	283.61 ± 54.96
(18)	47.55 ± 4.51	102.81 ± 44.56	538.12 ± 41.11	333.76 ± 16.30	130.56 ± 20.15	296.72 ± 23.89	36507.86 ± 3136.31	446.75 ± 40.19	468.76 ± 13.75	2634.11 ± 28.64	510.11 ± 1.20	53.45 ± 0.54	1.09 ± 0.62	861.32 ± 38.35	6.06 ± 1.02	588.75 ± 176.54
(19)	187.20 ± 6.50	50.26 ± 37.58	2489.21 ± 269.42	99.14 ± 11.08	72.50 ± 6.73	197.48 ± 0.38	41770.31 ± 3074.93	188.47 ± 45.86	313.85 ± 108.79	2636.29 ± 185.63	259.43 ± 25.26	61.69 ± 19.14	24.75 ± 9.54	3201.64 ± 375.22	10.89 ± 1.05	886.25 ± 111.68
(20)	76.51 ± 3.90	41.12 ± 23.32	2216.26 ± 73.06	126.95 ± 4.89	126.21 ± 55.76	538.49 ± 99.52	31934.09 ± 5671.24	165.11 ± 66.08	214.55 ± 25.99	1487.03 ± 60.99	55.17 ± 0.18	12.37 ± 0.89	8.55 ± 1.30	1016.05 ± 41.17	1.27 ± 0.10	758.02 ± 37.64
(21)	446.76 ± 25.53	57.25 ± 9.58	1168.16 ± 128.28	61.14 ± 9.41	87.81 ± 10.21	347.76 ± 30.71	22432.59** ± 1645.32**	413.32 ± 90.54	38.48 ± 15.85	692.10 ± 147.42	62.15 ± 4.92	5.70 ± 2.42	42.91 ± 1.36	255.81 ± 3.14	3.60 ± 0.46	223.65 ± 29.12
(22)	92.45 ± 5.24	32.03 ± 26.82	1243.93 ± 68.47	95.23 ± 27.07	104.22 ± 7.05	156.76 ± 71.92	33521.91 ± 1158.88	40.78 ± 4.50	231.70 ± 26.09	1309.25 ± 113.81	168.22 ± 10.83	4.80 ± 1.97	17.32 ± 0.42	538.37** ± 7.35**	1.84 ± 0.33	345.94 ± 99.73
(23)	99.88 ± 6.91	19.14 ± 2.25	1523.70 ± 44.43	80.24 ± 2.82	84.83 ± 7.94	207.32 ± 28.05	26628.08 ± 1083.66	84.44 ± 14.88	262.85 ± 0.67	935.29 ± 20.09	292.61 ± 5.64	9.96 ± 1.96	17.05 ± 3.82	520.98 ± 3.89	1.71 ± 0.71	477.32 ± 75.58
(24)	505.76 ± 5.04	204.29 ± 21.43	801.72 ± 43.45	174.58 ± 4.73	84.42 ± 11.77	374.36 ± 25.38	32871.64 ± 2578.00	331.90 ± 26.43	13.21 ± 2.63	725.86 ± 7.74	53.45 ± 2.62	9.85 ± 1.66	12.80 ± 1.15	445.52 ± 1.57	2.37 ± 0.22	234.18 ± 14.11
(25)	145.45 ± 4.75	97.76 ± 17.16	824.68 ± 86.31	273.32 ± 65.14	261.94 ± 3.10	907.44 ± 17.49	37252.14 ± 3375.34	83.65 ± 26.13	68.10 ± 13.80	1121.46 ± 45.12	276.90 ± 1.61	30.25 ± 8.27	1.49 ± 0.80	623.91 ± 12.00	4.80 ± 0.04	417.76 ± 85.03

ND: not detected; RHSW: raw *Polygonum multiflorum* Thunb.

**Table 2:** The content of 16 components in 21 batches of PHSW (*μ*g/g).

Number	Gallic acid	Procyanidins B1	Catechin	Gallate	Aloe-emodin glycoside	Polydatin	Stilbene glucoside	Rhaponticin	Resveratrol	Emodin glycoside	Physcion glycoside	Aloe emodin	Rhein	Emodin	Chrysophanol	Physcion
(1)	109.80 ± 32.65	76.59 ± 5.22	125.17 ± 12.60	8.36 ± 2.78	4.42 ± 0.93	53.68 ± 9.28	4721.66 ± 18.35	6.24 ± 0.42	2.29 ± 0.28	100.59 ± 8.88	27.11 ± 0.37	12.07 ± 1.48	11.85 ± 1.01	6.56 ± 4.51	5.38 ± 3.14	41.37 ± 14.04
(2)	713.20 ± 296.65	101.24 ± 6.73	155.08 ± 25.48	43.37 ± 12.77	38.00 ± 15.73	80.26 ± 16.94	22450.61 ± 2524.38	101.87 ± 30.91	10.07 ± 1.59	384.11 ± 49.88	69.78 ± 16.11	9.23 ± 0.08	1.24 ± 0.29	1514.16 ± 39.05	6.40 ± 2.06	186.75 ± 98.17
(3)	140.00 ± 61.24	38.18 ± 13.47	42.72 ± 1.86	5.00 ± 0.87	8.97 ± 0.30	4.79 ± 1.35	1298.17 ± 122.17	48.71 ± 27.22	4.26 ± 0.59	13.63 ± 2.12	4.14 ± 2.01	3.86 ± 1.61	3.45 ± 0.82	35.94 ± 8.66	8.55 ± 0.79	25.08 ± 5.29
(4)	149.90 ± 17.98	140.14 ± 42.10	223.22 ± 93.96	272.98 ± 94.78	15.22 ± 6.31	38.39 ± 13.61	10750.77 ± 3993.04	9.34 ± 1.79	1.65 ± 0.63	392.36 ± 16.08	171.50 ± 67.92	20.32 ± 7.03	4.07 ± 1.21	45.31 ± 9.47	4.16 ± 0.97	40.93 ± 9.52
(5)	155.34 ± 7.04	166.01 ± 2.45	190.60 ± 5.39	33.60 ± 7.58	19.12 ± 3.42	5.77 ± 3.49	7900.81 ± 29.84	10.96 ± 4.16	1.35 ± 0.17	134.30 ± 1.40	75.57 ± 0.22	14.09 ± 0.48	3.22 ± 0.04	20.70 ± 5.44	3.31 ± 0.25	31.61 ± 6.56
(6)	360.25 ± 10.36	15.35 ± 6.18	190.95 ± 53.46	56.34 ± 12.81	23.77 ± 8.31	67.90 ± 11.63	14897.07 ± 3894.91	47.39 ± 17.90	5.37 ± 1.31	306.74 ± 110.23	112.19 ± 47.53	11.58 ± 3.81	4.36 ± 2.20	227.13 ± 0.31	4.25 ± 0.92	81.56 ± 40.18
(7)	438.47 ± 146.29	140.21 ± 23.67	282.84 ± 18.00	21.76 ± 0.59	37.33 ± 13.90	84.15 ± 33.86	14055.11 ± 2827.67	17.11 ± 5.74	4.78 ± 1.12	194.31 ± 50.27	99.24 ± 23.23	29.31 ± 10.89	5.13 ± 0.89	188.79 ± 71.00	3.91 ± 0.57	88.77 ± 19.96
(8)	1308.68 ± 30.33	51.46 ± 14.12	239.51 ± 24.72	3.67 ± 1.29	13.57 ± 6.34	39.94 ± 26.64	17479.03 ± 284.81	71.22 ± 18.54	10.67 ± 2.16	302.61 ± 10.65	104.94 ± 2.79	13.62 ± 1.05	3.36 ± 1.42	1874.15 ± 119.89	5.91 ± 1.14	276.80 ± 80.31
(9)	417.09 ± 6.42	473.65 ± 97.15	498.61 ± 40.82	4.85 ± 0.64	29.92 ± 8.41	62.49 ± 38.18	15766.52 ± 54.78	51.29 ± 13.23	3.99 ± 1.72	305.14 ± 6.77	152.78 ± 3.47	11.77 ± 1.34	2.21 ± 0.89	424.31 ± 55.50	4.62 ± 0.77	134.62 ± 30.38
(10)	413.93 ± 89.78	59.44 ± 20.62	121.50 ± 33.04	162.62 ± 53.25	33.01 ± 12.78	21.25 ± 14.76	9118.58 ± 206.36	36.99 ± 16.49	1.86 ± 0.68	74.86 ± 8.04	35.15 ± 10.51	8.89 ± 0.39	2.01 ± 0.92	220.69 ± 18.72	1.78 ± 0.46	76.08 ± 7.62
(11)	156.33 ± 8.82	80.02 ± 4.60	79.60 ± 3.03	52.95 ± 7.20	17.82 ± 5.73	27.84 ± 3.49	2832.37 ± 132.73	22.06 ± 0.54	2.45 ± 0.18	111.99 ± 3.44	48.40 ± 0.58	6.76 ± 2.90	3.03 ± 0.93	38.46 ± 2.28	4.34 ± 0.27	39.08 ± 8.86
(12)	483.97 ± 25.73	371.92 ± 46.67	634.61 ± 88.24	30.37 ± 6.15	9.35 ± 1.20	51.31 ± 4.56	25203.07 ± 1959.70	121.63 ± 9.15	6.69 ± 0.45	132.91 ± 9.82	42.38 ± 8.28	3.42 ± 0.92	2.58 ± 0.37	919.49 ± 19.78	5.66 ± 1.99	210.62 ± 39.18
(13)	376.94 ± 51.89	213.30 ± 15.63	586.44 ± 8.93	9.26 ± 1.41	27.97 ± 17.22	132.39 ± 8.61	15026.59 ± 191.58	70.81 ± 15.66	5.33 ± 1.76	243.86 ± 6.29	142.19 ± 4.37	13.84 ± 2.88	2.52 ± 0.74	55.35 ± 1.62	4.24 ± 1.23	42.62 ± 3.36
(14)	525.92 ± 51.16	71.29 ± 3.18	154.50 ± 21.49	10.06 ± 6.94	56.41 ± 4.63	78.78 ± 1.57	16632.32 ± 87.69	21.11 ± 15.74	8.80 ± 2.32	176.07 ± 4.90	85.59 ± 4.43	9.80 ± 1.47	3.28 ± 0.67	278.56 ± 3.41	8.42 ± 1.03	113.28 ± 17.11
(15)	197.60 ± 10.72	284.87 ± 21.42	98.28 ± 5.26	9.38 ± 0.69	9.91 ± 0.79	10.50 ± 1.08	822.38 ± 45.06	41.83 ± 2.03	1.22 ± 0.03	1.11 ± 0.08	0.59 ± 0.06	2.05 ± 0.27	3.69 ± 0.34	13.61 ± 1.92	4.79 ± 0.11	33.82 ± 6.62
(16)	372.34 ± 0.38	322.74 ± 22.11	226.37 ± 2.84	5.97 ± 0.39	43.31 ± 3.42	49.55 ± 5.48	12730.59 ± 32.33	16.00 ± 1.95	5.01 ± 0.26	322.56 ± 0.26	167.01 ± 0.77	20.14 ± 0.70	1.14 ± 0.06	181.05 ± 29.11	181.05 ± 29.11	151.22 ± 9.79
(17)	451.70 ± 10.04	162.93 ± 6.56	329.48 ± 19.11	66.08 ± 3.19	72.43 ± 8.09	98.21 ± 7.08	15004.11 ± 57.69	57.96 ± 7.85	5.77 ± 0.83	546.16 ± 33.62	257.20 ± 9.22	25.13 ± 4.71	6.13 ± 3.62	174.52 ± 5.91	4.94 ± 0.55	71.92 ± 7.17
(18)	1101.01 ± 1.66	426.53 ± 34.47	352.63 ± 29.87	9.39 ± 0.58	31.17 ± 14.21	16.41 ± 1.36	1743.68 ± 2.63	24.07 ± 8.52	11.91 ± 3.76	13.81 ± 5.87	6.89 ± 1.70	5.79 ± 2.58	3.10 ± 0.36	35.73 ± 0.31	4.71 ± 0.28	60.58 ± 7.89
(19)	334.67 ± 28.89	83.18 ± 37.39	174.31 ± 19.48	15.88 ± 0.91	44.82 ± 8.48	57.30 ± 5.42	13295.14 ± 684.33	45.28 ± 12.65	9.02 ± 2.33	204.47 ± 24.02	102.15 ± 9.26	12.09 ± 1.47	3.60 ± 0.36	372.31 ± 23.06	6.92 ± 2.74	118.48 ± 9.30
(20)	711.09 ± 0.18	57.15 ± 18.18	252.66 ± 15.05	6.95 ± 0.30	61.25 ± 22.67	89.23 ± 23.5.57	11682.62 ± 273.11	42.77 ± 4.82	6.94 ± 1.98	127.27 ± 2.29	70.44 ± 4.07	28.77 ± 6.23	3.02 ± 1.79	153.29 ± 4.57	4.35 ± 0.22	87.93 ± 16.40
(21)	426.66 ± 0.70	211.31 ± 2.95	400.09 ± 6.61	8.87 ± 0.37	12.82 ± 6.98	112.61 ± 7.62	15600.62 ± 134.76	25.60 ± 3.37	4.78 ± 0.23	128.93 ± 1.21	71.56 ± 7.07	21.25 ± 4.33	5.87 ± 1.68	82.53 ± 4.16	5.29 ± 0.34	68.26 ± 4.60

PHSW: processed *Polygonum multiflorum* Thunb.

**Table 3:** Fingerprint similarity for 25 batches of RHSW.

Number	Collection location	Similarity degree
(1)	Kunming, Yunnan	0.48
(2)	Changchun, Jilin	0.995
(3)	Tongrentang, Anhui	0.999
(4)	Tongrentang, Beijing	0.994
(5)	Dabashan, Sichuan	0.985
(6)	Bozhou, Anhui	0.995
(7)	Huoshan, Anhui	0.99
(8)	Yuncheng, Shanxi	0.993
(9)	Shantou. Guangzhou	0.997
(10)	Zhaoqing, Guangzhou	0.994
(11)	Yulin, Guangxi	0.999
(12)	Yulinhong, Guangxi	0.977
(13)	Yancheng, Jiangsu	0.67
(14)	Baoding, Heibei	0.953
(15)	Shijiazhuang, Heibei	0.99
(16)	Taihang, Henan	0.997
(17)	Yichang, Hubei	0.971
(18)	Xichang, Sichuan	0.987
(19)	Leping, Guizhou	0.982
(20)	Liupanshui, Guizhou	0.99
(21)	Wangmo, Guizhou	0.976
(22)	Zhenping, Guizhou	0.994
(23)	Qianxi, Guizhou	0.993
(24)	Qianjiang, Chongqing	0.994
(25)	Baoji, Shanxi	0.997

RHSW: raw *Polygonum multiflorum* Thunb.

**Table 4:** Principal component analysis of 25 batches of RHSW.

Principal component	Eigenvalue	Variance contribution rate/%	Cumulative contribution rate/%
*F*_1_	5.096	31.852	31.852
*F*_2_	2.741	17.134	48.986
*F*_3_	1.722	10.763	59.749
*F*_4_	1.431	8.942	68.691
*F*_5_	1.238	7.738	76.429
*F*_6_	0.944	5.901	82.33

RHSW: raw *Polygonum multiflorum* Thunb.

**Table 5:** Component loading matrix of 25 batches of RHSW.

Component	*F*_1_	*F*_2_	*F*_3_	*F*_4_	*F*_5_	*F*_6_
Gallic acid	−0.488	−0.037	0.188	0.431	−0.035	0.679
Procyanidins B1	0.06	0.206	−0.413	0.736	0.177	−0.159
Catechin	−0.068	0.254	0.671	0.249	0.383	0.013
Gallate	−0.054	0.814	−0.193	−0.049	−0.092	−0.064
Aloe-emodin glycosides	−0.01	0.92	0.028	0.003	−0.208	−0.149
Polydatin	−0.075	0.911	0.096	−0.021	−0.222	0.048
Stilbene glucoside	0.69	0.225	−0.104	−0.13	−0.185	0.481
Rhaponticin	0.58	0.246	−0.432	0.253	0.362	0.258
Resveratrol	0.814	0.141	0.138	−0.2	0.083	0.062
Emodin glycosides	0.885	0.011	−0.052	−0.106	0.159	0.105
Physcion glycosides	0.768	−0.092	−0.483	−0.18	−0.011	−0.024
Aloe emodin	0.594	−0.286	−0.178	0.351	−0.158	−0.079
Rhein	0.314	0.241	0.257	−0.121	0.758	−0.094
Emodin	0.711	−0.074	0.539	0.104	−0.303	0.087
Chrysophanol	0.588	−0.054	0.25	0.566	−0.235	−0.289
Physcion	0.83	−0.079	0.34	−0.073	−0.116	−0.099

RHSW: raw *Polygonum multiflorum* Thunb.

**Table 6:** Integrated *F* value of 25 batches of RHSW.

Number	*F*_1_	*F*_2_	*F*_3_	*F*_4_	*F*_5_	*F*_6_	Integrated *F* value
(1)	0.24	35.53	54.85	22.19	30.94	−0.04	16.44
(2)	616.25	267.29	−117.87	−189.07	−276.41	908.37	244.71
(3)	551.02	244.74	−95.77	−174.65	−261.36	833.98	220.51
(4)	291.29	132.01	−31.00	−91.90	−147.79	425.47	117.52
(5)	796.44	325.50	−151.70	−242.11	−331.64	1095.05	310.43
(6)	687.24	309.49	−145.33	−207.93	−304.70	983.88	272.18
(7)	546.77	304.84	−105.02	−181.52	−284.62	859.42	227.54
(8)	524.40	254.21	−75.06	−164.60	−270.25	816.93	215.09
(9)	494.95	219.97	−73.63	−155.45	−247.63	773.87	200.02
(10)	481.93	213.70	−94.76	−154.16	−227.15	755.45	193.14
(11)	493.75	224.95	−85.81	−152.93	−233.84	746.15	198.84
(12)	289.63	174.38	25.99	−51.06	−89.70	467.44	141.01
(13)	−0.27	2.44	2.43	2.76	2.25	−7.73	0.56
(14)	176.07	114.28	11.25	−28.73	−45.98	252.08	85.62
(15)	524.26	211.64	−111.53	−168.12	−234.83	741.38	201.79
(16)	398.24	169.72	−69.66	−123.94	−201.44	607.49	157.61
(17)	137.05	55.14	−20.87	−41.88	−71.38	203.74	53.61
(18)	656.90	270.83	−127.48	−210.59	−286.18	913.07	254.82
(19)	768.42	303.84	−35.08	−227.74	−331.74	1062.93	309.73
(20)	552.91	252.52	−43.32	−164.41	−237.30	801.13	228.93
(21)	366.33	176.11	−56.11	−103.16	−162.39	569.10	152.61
(22)	559.68	245.12	−89.96	−179.89	−257.44	840.30	224.16
(23)	452.15	200.85	−55.84	−139.17	−198.19	667.13	184.01
(24)	527.36	245.34	−104.34	−160.03	−259.82	829.94	213.34
(25)	613.40	294.90	−115.27	−203.22	−308.50	931.42	246.42

RHSW: raw *Polygonum multiflorum* Thunb.

**Table 7:** Weight factor of component to the whole RHSW.

Component	*F*1	*F*2	*F*3	*F*4	*F*5	*F*6	*W*_*t*_	*W*	% of weight
Gallic acid	−0.216	−0.022	0.143	0.360	−0.032	0.699	1.376	147.360	0.933
Procyanidins B_1_	0.027	0.124	−0.315	0.615	0.159	−0.164	5.359		3.637
Catechin	−0.030	0.153	0.511	0.208	0.344	0.013	11.777		7.992
Gallate	−0.024	0.492	−0.147	−0.041	−0.083	−0.066	4.685		3.179
Aloe-emodin glycoside	−0.004	0.556	0.021	0.003	−0.187	−0.153	7.281		4.941
Polydatin	−0.033	0.550	0.073	−0.018	−0.200	0.049	7.750		5.259
Stilbene glucoside	0.306	0.136	−0.079	−0.109	−0.166	0.495	11.875		8.058
Rhaponticin	0.257	0.149	−0.329	0.212	0.325	0.266	13.161		8.931
Resveratrol	0.361	0.085	0.105	−0.167	0.075	0.064	13.537		9.186
Emodin glycosides	0.392	0.007	−0.040	−0.089	0.143	0.108	13.124		8.906
Physcion glycosides	0.340	−0.056	−0.368	−0.151	−0.010	−0.025	4.353		2.954
Aloe emodin	0.263	−0.173	−0.136	0.293	−0.142	−0.081	5.007		3.398
Rhein	0.139	0.146	0.196	−0.101	0.681	−0.097	12.830		8.707
Emodin	0.315	−0.045	0.411	0.087	−0.272	0.090	12.886		8.745
Chrysophanol	0.261	−0.033	0.191	0.473	−0.211	−0.297	10.630		7.214
Physcion	0.368	−0.048	0.259	−0.061	−0.104	−0.102	11.730		7.960

RHSW: raw *Polygonum multiflorum* Thunb.

## Additional Points

The supporting information is the resource of RHSW and PHSW; the method validation of RHSW and PHSW; the analytical procedure and HCA analysis of PHSW.
