# Supplementary material for: A New Strategy for Quality Evaluation and Identification of Representative Chemical Components in Polygonum multiflorum Thunb
Source: Evid Based Complement Alternat Med. 2017 Jan 24;2017:6238464. doi: 10.1155/2017/6238464 (PMC5294750; doi:10.1155/2017/6238464)

Table S1 RHSW and PHSW from various productive area

RHSW	productive area	PHSW	productive area
1	Kunming, Yunnan Province	1	Yulin, Guangxi Province
2	Changchun, Jilin Province	2	Baoding, Hebei Province
3	Tongrentang, Anhui Province	3	Guangzhou, Guangdog Province
4	Tongrentang, Beijing	4	Zhaoqing, Guangdong Province
5	Dabashan, Sichuan Province	5	Bozhou, Anhui Province
6	Bozhou, Anhui Province	6	Shaoyang, HunanProvince
7	Huoshan, Anhui Province	7	Shijiazhuang, Hebei Province
8	Yuncheng, Shanxi Province	8	Changsha, Hunan Province
9	Shantou, Guangdong Province	9	Xian Shaanxi Province
10	Zhaoqing, Guangdong Province	10	Suzhou, Jiangsu Province
11	Yulin, Guangxi Province	11	Handan, Hebei Province
12	Yulihong, Guangxi Province	12	Dabashan, Sichuan Province
13	Yancheng, jiangsu Province	13	Heyuan, Guangdong Province
14	Baoding, Hebei Province	14	Tongrentang, Beijing
15	Shijiazhuang, Hebei Province	15	Nantong, Jiangsu Province
16	Taihang, Henan Province	16	Weinan, Shaanxi Province
17	Yichang, Hubei Province	17	Dazhou, Sichuan Province
18	Xichuang, Sichuan Province	18	Guiyang,Guizhou Province
19	Leping, Guizhou Province	19	Hangzhou, Zhejiang Province
20	Liupanshui, Guizhou Province	20	Leshan, Sichuan Province
21	Wangmo, Guizhou Province	21	Fuzhou, Fujian Province
22	Zhenning,Guizhou Province		
23	Qianxi,Guizhou Province		
24	Qianjiang,Guizhou Province		
25	Baojin, Shaanxi Province		

RHSW : Raw *Polygonum multiflorum* Thunb.; PHSW: Processed *Polygonum multiflorum* Thunb.

Table S2 The calibration curve range for 16 components in HSW

Component	Regression equation	R^2	Linearity range μg/mL
Gallic acid	y = 92.84x + 0.04652	0.9998	0.33～32.75
Procyanidins B_1	y = 12.091x + 4.093	0.9995	0.33～32.67
Catechin	y = 18.738x + 11.358	0.9997	0.39～196.00
Gallate	y = 79.687x + 34.41	0.9992	0.71～71.00
Aloe-emodin glycoside	y = 31.66x + 4.313	0.9981	0.39～19.50
Polydatin	y = 43.21x + 21.93	0.9983	0.62～62.50

Stilbene glucoside	y = 22.77x + 2462.6	0.9994	2.70～2700.00
Rhaponticin	y = 34.648x + 9.4192	0.9999	0.24～35.50
Resveratrol	y = 83.18x + 17.867	0.9999	0.02～47.00
Emodin glycoside	y = 79.125x + 2.523	0.9999	1.88～375.00
Physcion glycoside	y = 108.7x - 44.554	0.9998	0.43～43.00
Aloe emodin	y = 46.95x + 6.665	0.9999	0.02～8.88
Rhein	y = 55.14x + 11.70	0.9976	0.01～8.10
Emodin	y = 127.59x - 399.87	0.9991	0.25～250.00
Chrysophanol	y = 66.495x + 18.005	0.9995	0.03～5.00
Physcion	y = 80.504x + 326.26	0.9995	0.22～44.00

HSW: *Polygonum multiflorum* Thunb.

Table S3 Intra- and inter-day precision for 16 components in HSW (mean±*s*，n=6)

Component	Concentration (μg/mL)	Intra-assay		Inter-assay	
		mean±*s*	RSD%	mean±*s*	RSD%
Gallic acid	4.1	4.04±0.02	0.50	4.08±0.01	0.25
	16.38	16.41±0.27	1.64	16.35±0.26	1.59
	32.75	32.67±0.65	1.99	32.56±0.58	1.78
Procyanidins B_1	2.41	2.38±0.01	0.42	2.36±0.03	1.27
	13.45	13.31±0.24	1.80	13.39±0.25	1.87
	38.5	38.77±0.35	0.90	38.34±0.45	1.17
Catechin	3.31	3.28±0.01	0.38	3.26±0.05	1.53
	47.90	47.77±0.35	0.73	47.68±0.56	1.17
	92.5	92.31±0.24	0.26	92.28±1.25	1.35
Gallate	2.54	2.47±0.02	0.94	2.46±0.04	1.63
	19.02	18.93±0.14	0.73	18.86±0.23	1.22
	35.5	35.31±0.63	1.78	35.36±0.45	1.27
Aloe-emodin glycoside	1.22	1.25±0.02	1.60	1.18±0.02	1.69
	5.48	5.37±0.06	1.12	5.39±0.08	1.48
	9.75	9.66±0.11	1.15	9.72±0.10	1.03
Polydatin	1.95	1.84±0.03	1.63	1.92±0.03	1.25
	16.6	16.36±0.27	1.65	16.42±0.15	0.91
	31.25	30.98±0.18	0.58	30.88±0.56	1.81
Stilbene glucoside	248	246.23±1.35	0.55	245.89±2.36	0.96
	1076	1074.59±1.56	0.15	1074.22±1.23	0.11
	4056	4053.21±2.36	0.06	4054.28±2.54	0.06
Rhaponticin	0.41	0.43±0.007	1.63	0.39±0.01	1.79
	8.88	8.82±0.09	1.07	8.79±0.11	1.25
	35.5	35.43±0.21	0.60	35.35±0.56	1.58

Resveratrol	1.47	1.37±0.02	1.46	1.42±0.02	1.41
	24.24	23.73±0.26	0.98	24.11±0.26	1.08
	47.00	46.90±0.73	1.55	46.78±0.88	1.88
Emodin glycosides	7.81	7.97±0.15	1.88	7.74±0.15	1.94
	62.50	61.27±0.17	0.27	60.89±1.17	1.92
	375.00	374.23±0.63	0.17	368.98±4.56	1.24
Physcion glycosides	0.67	0.60±0.01	1.57	0.62±0.01	1.61
	5.38	5.41±0.03	0.55	5.28±0.10	1.89
	43	41.92±0.23	0.55	42.22±0.56	1.33
Aloe emodin	0.28	0.26±0.005	1.92	0.25±0.01	2.00
	4.58	4.44±0.07	1.68	4.52±0.07	1.65
	8.88	8.89±0.17	1.91	8.78±0.12	1.37
Rhein	0.20	0.18±0.003	1.67	0.18±0.002	1.11
	7.41	7.38±0.11	1.49	7.35±0.09	1.22
	14.62	14.46±0.09	0.62	14.34±0.15	1.05
Emodin	15.62	15.11±0.26	1.69	15.28±0.12	0.79
	132.81	131.48±2.45	1.86	130.89±2.33	1.78
	250	248.53±2.89	1.16	248.25±2.25	0.91
Chrysophanol	0.22	0.17±0.003	1.76	0.19±0.003	1.58
	2.64	2.59±0.03	1.18	2.31±0.05	1.90
	5.06	5.03±0.03	0.59	4.95±0.06	1.21
Physcion	2.75	2.55±0.03	1.29	2.68±0.03	1.23
	23.36	23.31±0.16	0.69	22.56±0.20	0.89
	44	43.44±0.34	0.78	42.78±0.78	1.82

HSW: *Polygonum multiflorum* Thunb.

Table S4 Reproducibility and stability of 16 components in HSW(mean±*s*，n=6)

Component	Reproducibility (μg/mL)		Stability(μg/mL)	
	mean±*s*	RSD%	mean±*s*	RSD%
Gallic acid	7.69±0.02	0.27	7.64±0.14	1.90
Procyanidins B_1	15.46±0.08	0.55	15.64±0.13	0.84
Catechin	42.03±0.13	0.30	41.59±0.18	0.42
Gallate	2.06±0.03	1.23	2.05±0.04	1.95
Aloe-emodin glycoside	5.27±0.10	1.90	5.15±0.04	0.85
Polydatin	14.43±0.10	0.68	14.47±0.14	0.97
Stilbene glucoside	2113.30±22.12	1.05	2064.95±21.52	1.04
Rhaponticin	3.27±0.06	1.96	3.40±0.06	1.83
Resveratrol	17.54±0.01	0.07	17.05±0.26	1.55

Emodin glycoside	114.71±0.16	0.14	113.56±0.35	0.31
Physcion glycoside	1.68±0.02	1.19	1.62±0.01	0.62
Aloe emodin	4.44±0.03	0.68	4.64±0.05	1.39
Rhein	1.98±0.02	1.01	1.97±0.02	1.01
Emodin	94.87±0.42	0.44	95.89±0.46	0.48
Chrysophanol	5.11±0.08	1.57	4.89±0.02	0.42
Physcion	45.08±0.71	1.57	46.79±0.22	0.47

HSW: *Polygonum multiflorum* Thunb.

Table S5 Recovery of 16 components in HSW (mean±*s*，n=6)

Component	Sample (μg/mL)	Added (μg/mL)	Measured (μg/mL)	Recovery%
Gallic acid	3.83±0.04	4.1	7.74±0.09	95.27
		16.38	19.62±2.45	97.01
		32.75	35.11±1.89	95.98
Procyanidins B_1	7.78±0.05	2.41	12.98±3.78	103.02
		9.63	17.54±0.15	101.39
		13.45	20.03±2.87	94.36
Catechin	20.90±0.08	3.31	25.88±6.89	106.89
		23.13	42.46±0.18	93.20
		47.90	65.12±5.66	94.65
Gallate	1.03±0.02	1.27	2.25±0.02	96.32
		2.54	35.07±1.89	98.23
		19.02	21.10±1.34	105.23
Aloe-emodin glycosides	2.60±0.05	1.22	3.67±0.65	96.10
		2.44	5.18±0.06	105.56
		5.48	7.48±0.43	92.59
Polydatin	7.23±0.06	1.95	8.58±0.77	93.48
		7.81	14.55±0.08	93.78
		16.6	23.06±2.49	96.78
Stilbene glucoside	1044.56±10.91	248	237.67±14.88	93.12
		1014.00	2064.68±13.68	100.60
		4056	4812.89±45.634	94.36
Rhaponticin	1.67±0.03	0.41	2.00±0.68	96.25
		2.22	3.94±0.10	102.39
		8.88	10.42±1.35	98.72
Resveratrol	8.65±0.07	1.47	9.64±1.59	95.25
		5.88	15.05±0.09	108.89
		24.24	31.36±6.68	95.35
Emodin	57.07±0.13	7.81	59.83±3.41	92.22

glycosides		62.50	121.56±1.25	103.19
		375.00	395.60±14.98	91.56
Physcion glycosides	0.83±0.03	0.67	1.52±0.08	103.72
		5.38	5.73±1.04	92.36
		43	41.64±3.44	95.00
Aloe emodin	2.27±0.05	0.28	2.49±0.08	97.64
		2.22	4.44±0.04	97.69
		4.58	6.45±0.36	94.16
Rhein	0.99±0.04	0.20	1.17±0.12	98.22
		0.92	1.97±0.07	106.61
		7.41	7.83±0.72	93.25
Emodin	47.69±0.22	15.62	59.92±5.79	94.65
		41.88	91.89±1.17	105.54
		132.81	168.37±10.52	93.28
Chrysophanol	2.50±0.03	0.22	2.51±0.67	92.44
		2.53	4.89±0.01	94.49
		5.06	8.05±1.19	106.51
Physcion	22.97±0.23	2.75	28.18±0.53	109.58
		22.00	46.79±0.89	108.28
		44	62.72±1.73	93.66

HSW: *Polygonum multiflorum* Thunb.

Table S6 Fingerprint similarity for 21 batches PHSW

No	Collection location	Similarity degree	No	Collection location	Similarity degree
1	Yulin, Guangxi	0.999	12	Dabashan,Sichuan	0.996
2	Baoding, Heibei	0.972	13	Heyuan, Guangdong	0.998
3	Guangzhou, Guangdong	0.758	14	Tongrentang, Beijing	1
4	Zhaoqing ,Guangzhou	0.996	15	Nantong, Jiangsu	0.256
5	Bozhou, Anhui	0.652	16	Weinan, Shanxi	0.998
6	Shaoyang, Hunan	0.998	17	Dazhou, Sichuan	0.996
7	Shijiazhuang, Heibei	0.984	18	Guiyang, Guizhou	0.517
8	Changsha, Hunan	0.993	19	Hanzhou, Zhejiang	0.996
9	Xi'an, Shanxi	0.998	20	Leshan, Sichuan	0.889
10	Suzhou, Jiangsu	0.996	21	Fuzhou, Fujian	0.998
11	Handan, Hebei	0.99			

PHSW: Processed *Polygonum multiflorum* Thunb.

Table S7 Principal component analysis of 21 batches PHSW

Principal component	Eigenvalue	Variance contribution rate / %	Cumulative contribution rate / %
F1	5.16	32.25	32.25
F2	3.28	20.47	52.73
F3	1.76	11.00	63.73
F4	1.61	10.06	73.79
F5	1.19	7.46	81.25

PHSW: Processed *Polygonum multiflorum* Thunb.

Table S8 Component loading matrix of 21 batches PHSW

Component	F_1	F_2	F_3	F_4	F_5
Gallic acid	0.73	-0.36	0.13	0.03	-0.36
Procyanidins B_1	0.15	-0.09	-0.86	0.16	-0.18
Catechin	0.56	0.15	-0.68	0.29	0.17
Gallate	-0.22	0.39	0.01	-0.74	0.01
Aloe-emodin glycosides	0.41	0.50	0.25	0.23	-0.53
Polydatin	0.53	0.55	-0.01	0.44	0.24
Stilbene glucoside	0.87	0.17	-0.09	-0.11	0.29
Rhaponticin	0.70	-0.37	-0.16	-0.16	0.28
Resveratrol	0.74	-0.33	0.25	0.29	-0.31
Emodin glycosides	0.58	0.63	0.14	-0.31	0.11
Physcion glycosides	0.44	0.79	-0.03	-0.14	0.00
Aloe emodin	0.20	0.80	0.18	0.16	-0.14
Rhein	-0.35	0.25	0.29	0.38	0.54
Emodin	0.76	-0.40	0.22	-0.35	0.15
Chrysophanol	0.23	-0.42	0.45	0.37	0.26
Physcion	0.83	-0.30	0.06	-0.23	0.05

PHSW: Processed *Polygonum multiflorum* Thunb.

Table S9 Integrated F value of 21 batches PHSW

No	F_1	F_2	F_3	F_4	F_5	integrated F value
1	96.50	23.58	-20.64	-19.94	63.73	36.52
2	479.95	83.81	-66.01	-125.46	303.13	175.15
3	30.38	3.62	-5.51	-5.89	15.62	10.54
4	217.81	61.22	-44.12	-58.29	145.87	83.16
5	159.27	38.76	-35.88	-35.30	104.97	59.79
6	305.06	69.29	-51.21	-72.75	198.76	114.75

7	289.44	64.04	-55.21	-63.32	185.03	108.10
8	399.06	51.27	-44.21	-106.65	232.14	141.32
9	330.92	71.56	-75.57	-72.26	211.63	121.91
10	189.15	38.09	-31.88	-47.80	118.00	69.48
11	62.18	14.27	-12.33	-14.46	36.11	22.92
12	521.47	100.34	-104.88	-120.16	341.99	191.12
13	310.22	73.21	-70.35	-61.80	201.56	116.42
14	340.28	73.48	-61.56	-76.03	220.69	127.17
15	22.94	0.87	-13.48	-0.73	6.19	6.50
16	264.72	61.32	-54.87	-59.66	167.81	98.67
17	313.52	80.03	-58.74	-73.02	200.77	118.98
18	59.62	-2.80	-22.51	-0.74	4.92	16.53
19	276.97	57.36	-46.23	-65.09	175.97	102.84
20	244.61	49.82	-42.63	-51.18	148.64	90.58
21	315.88	71.47	-67.76	-65.49	207.75	118.27

PHSW: Processed *Polygonum multiflorum* Thunb.

Table S10 Weight factor of component to the whole PHSW

Component	A1	A2	A3	A4	A5	Wt	W	% of weight
Gallic acid	0.32	-0.20	0.10	0.02	-0.33	5.15	131.49	3.92
Procyanidins B1	0.07	-0.05	-0.65	0.13	-0.16	-5.98		-4.55
Catechin	0.25	0.08	-0.51	0.23	0.16	7.47		5.68
Gallate	-0.10	0.22	0.01	-0.58	0.01	-4.43		-3.37
Aloe-emodin glycoside	0.18	0.28	0.19	0.18	-0.49	11.75		8.94
Polydatin	0.23	0.30	-0.01	0.35	0.22	18.79		14.29
Stilbene glucoside	0.38	0.09	-0.07	-0.09	0.27	14.64		11.13
Rhaponticin	0.31	-0.20	-0.12	-0.13	0.26	5.07		3.86
Resveratrol	0.33	-0.18	0.19	0.23	-0.28	9.03		6.87
Emodin glycoside	0.26	0.35	0.11	-0.24	0.10	14.81		11.27
Physcion glycoside	0.19	0.44	-0.02	-0.11	0.00	13.82		10.51
Aloe emodin	0.09	0.44	0.14	0.13	-0.13	13.69		10.41
Rhein	-0.15	0.14	0.22	0.30	0.49	6.96		5.29
Emodin	0.33	-0.22	0.17	-0.28	0.14	6.34		4.82
Chrysophanol	0.10	-0.23	0.34	0.29	0.24	6.96		5.29
Physcion	0.37	-0.17	0.05	-0.18	0.05	7.41		5.63

PHSW: Processed *Polygonum multiflorum* Thunb.

Fig.S1 HCA analysis of PHSW decoction pieces with 16 components

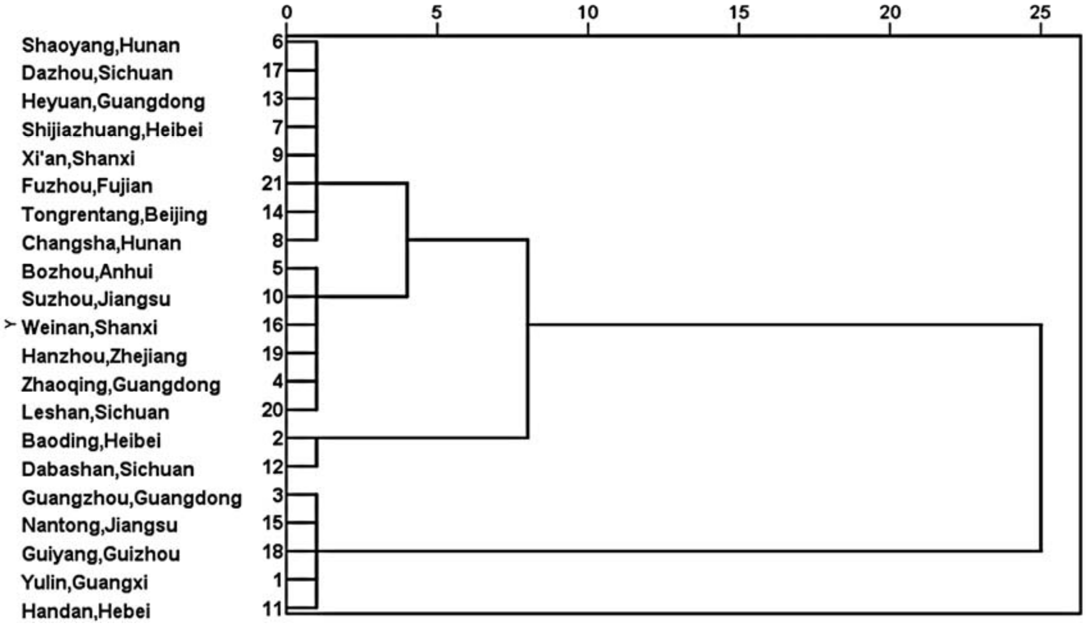

Fig.S2 HCA analysis of PHSW decoction pieces with selected 6 representative components

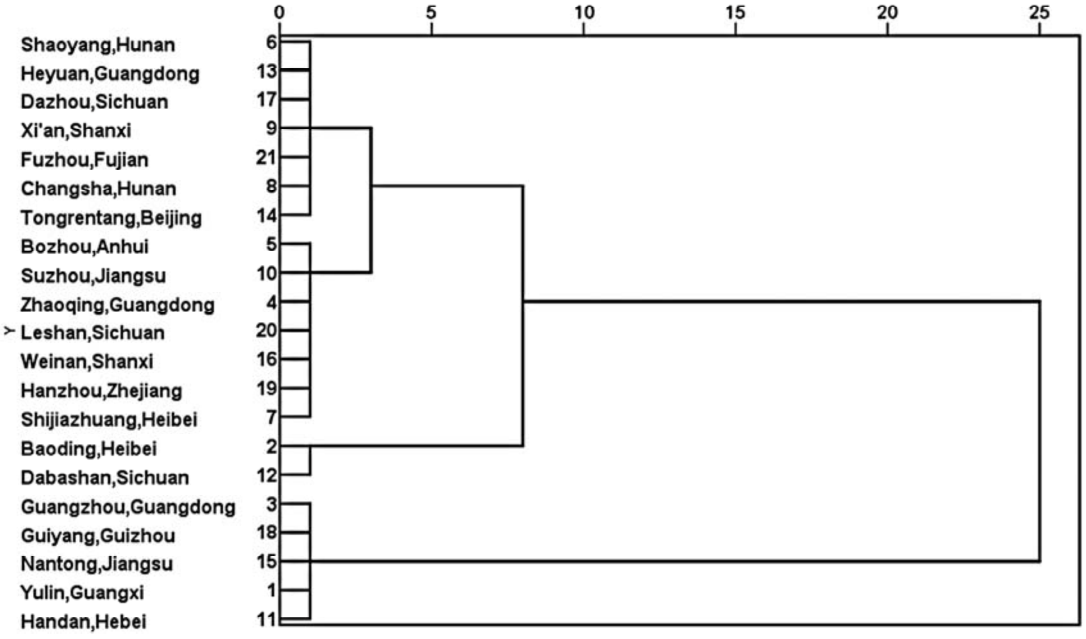

Supplement: Supplementary file 1 — The supporting materials were showed in supplementary tables and figures. The various productive areas of Raw Polygonum multiflorum Thunb. and Processed Polygonum multiflorum Thunb. were included in Table S1. The method validation results of the established HPLC method in detecting various components in Polygonum multiflorum Thunb. were showed in Table S2~5. Also, the calculation results in relation to Processed Polygonum multiflorum Thunb. were showed in Table S6~10. In addition, HCA analysis of Processed Polygonum multiflorum Thunb. decoction pieces with 16 components were showed in Figure S1 and HCA analysis of Processed Polygonum multiflorum Thunb. decoction pieces with selected 6 representative components were showed in Figure S2. [file 6238464.f1.pdf]
